# Gonadotropin-Induced Spermatogenesis in CHH Patients with Cryptorchidism

**DOI:** 10.1155/2019/6743489

**Published:** 2019-12-18

**Authors:** Zhaoxiang Liu, Jiangfeng Mao, Hongli Xu, Xi Wang, Bingkun Huang, Junjie Zheng, Min Nie, Hongbing Zhang, Xueyan Wu

**Affiliations:** ^1^Key Laboratory of Endocrinology, Department of Endocrinology, Peking Union Medical College Hospital, National Health Commission of People's Republic of China, Beijing, China; ^2^Beijing Tsinghua Changgung Hospital, School of Clinical Medicine, Tsinghua University, Beijing, China; ^3^Department of Physiology, State Key Laboratory of Medical Molecular Biology, Institute of Basic Medical Sciences and School of Basic Medicine, Peking Union Medical College and Chinese Academy of Medical Sciences, Beijing, China

## Abstract

Congenital hypogonadotropic hypogonadism (CHH) patients with cryptorchidism history usually have poor spermatogenesis outcome, while researches focusing on this population are rare. This study retrospectively evaluated gonadotropin-induced spermatogenesis outcome in CHH patients with cryptorchidism (*n* = 40). One hundred and eighty-three CHH patients without cryptorchidism were served as control. All patients received combined gonadotropins therapy (HCG and HMG) and were followed up for at least 6 months. The median follow-up period was 24 (15, 33) months (totally 960 person-months). Sperm (>0/ml) initially appeared in semen at a median of estimated 24 months (95% confidence interval (CI) 17.8–30.2). Twenty (20/40, 50%) patients succeeded in producing sperms, and the average time to produce first sperm was 19 ± 8 months. Five pregnancies were achieved in 9 (5/9, 56%) couples who desired for children. Compared with CHH patients without cryptorchidism (*n* = 183), cryptorchid patients had longer median time for sperm appearance in semen (24 months vs. 15 months, *P* < 0.001), lower rate of spermatogenesis (50% vs. 67%, *P*=0.032), and lower mean sperm concentration (1.9 (0.5, 8.6) million/ml vs. 11.1(1.0, 25.0) million/ml, *P*=0.006) at the last visit. In conclusion, CHH patients with cryptorchidism require a longer period for gonadotropin-induced spermatogenesis. The successful rate and sperm concentration were lower than patients without cryptorchidism.

## 1. Introduction

Congenital hypogonadotropic hypogonadism (CHH), caused by GnRH deficiency or dysfunction, is characterized by delayed or absent puberty, development, and infertility. When fertility is desired, pulsatile GnRH infusion or combined human chorionic gonadotropin (HCG) and human menopausal gonadotropin (HMG) therapy can be used for spermatogenesis.

Cryptorchidism (particularly bilateral) has a significant negative impact on fertility potential [1, 2]. Compared with 1–3% cryptorchidism rate in full-term healthy neonates [3], the prevalence of cryptorchidism in CHH patients is about 10–20% [4, 5]. For normal male fetuses, testosterone and insulin-like factor 3 (INSL3) secreted by Leydig cells are primary regulators for testicular descent [6]. However, the normal procedure of testicular descent is disturbed due to a low level of testosterone and impaired Leydig cell function in CHH patients [7]. Abnormal position exposes testes to hyperthermia which impairs gonocyte transformation and subsequent mature cessation [8]. Therefore, it is recommended that cryptorchidism should be treated within one year after birth, hoping to maximally preserve the spermatogenic potentiality [9].

Cryptorchidism has been referred as a detrimental factor for producing sperms in CHH patients [4, 10–12]. In this study, we report the fertility and paternity outcomes in 40 CHH patients with cryptorchidism treated with gonadotropins (HCG/HMG).

## 2. Materials and Methods

### 2.1. Subjects

This retrospective cohort study included 40 CHH patients with cryptorchidism histories referred to our hospital between 2005 and 2014. One hundred and eighty-three CHH patients without cryptorchidism treated with HCG/HMG were served as control (noncryptorchidism group). The diagnosis of CHH was made according to the criteria reported previously [13]. Cryptorchidism was defined as one or both testes remained undescended 6 months after birth.

The study protocol was reviewed and approved by the Ethics Committee of the Peking Union Medical College Hospital, and all aspects of the study comply with the Declaration of Helsinki. Ethics Committee of the Peking Union Medical College Hospital approved that no informed consent was required because data were analyzed retrospectively and anonymously.

### 2.2. Treatment and Data Collection

Patients discontinued androgen therapy (if used) for at least 3 months before starting gonadotropins therapy. Combined HCG (2000 U, Livzon Pharmaceutical Co, Guangdong, China) and HMG (75 U, Livzon Pharmaceutical Co.) was intramuscularly injected twice weekly at the beginning. The dosages of gonadotropins were adjusted according to serum testosterone level and sperm output. HCG dosage would increase to 2500–5000 U to maintain a serum testosterone level at 10–20 nmol/L. If sperm did not appear in seminal fluid after treatment of 6 months, HMG dosage would increase to 150 U. All patients were treated with gonadotropins for at least 6 months. Regular follow-ups were conducted at an interval of 3–6 months.

All patients were tested for their sense of smell. We prepared alcohol, vinegar, and water. If the patient could not distinguish them, he would be diagnosed as Kallmann syndrome preliminarily. Each patient had MR for pituitary and olfactory bulb and tract. Secondary sexual characteristics, testicular volumes, serum gonadotropins, serum testosterone, and sperm count were measured on each visit. For undescended testes, the testis volume was defined as 0. For descended testis (natural or after orchidopexy), the testicular volume was measured using a Prader orchidometer. The mean value of bilateral testicular volumes was used in data analysis. Semen samples were collected by masturbation and were analyzed according to the standard World Health Organization method [14]. Follicle-stimulating hormone (FSH), luteinizing hormone (LH), and total testosterone levels were measured using commercial kits by the chemiluminescent method (ACS 180 Automatic Chemiluminescence System; Bayer, Germany). The intra- and interassay coefficients of variation of total testosterone were 5.6% and 6.6%, respectively. All the patients were intramuscularly injected with triptorelin (100 *μ*g) at the first visit, and basal and peak LH levels were measured [15]. Sperm motility was classified as fast progressive sperm (*A*), slow progressive sperm (*B*), nonprogressive sperm, (*C*) and immotile sperm (*D*). The proportion in each motility categories was assessed.

### 2.3. Outcomes

The primary outcome was defined as the first sperm detection under microscopy (the semen were centrifugated if necessary). The secondary outcome was paternity. Self-reported pregnancy of the partners was noted.

### 2.4. Statistical Analysis

SPSS version 17.0 was used for data analysis. Normal distributive data were expressed as the mean ± SD, and nonnormal distributive data were expressed as median (quartiles). The paired *t*-test was used to compare the plasma testosterone and testicular volumes before and after the treatment. Kaplan–Meier analyses were used to estimate the median time to achieve different sperm thresholds. Cox regression models were built to analyze the predictors of successful spermatogenesis. *χ*^2^ test was used to compare the differences between cryptorchidism and noncryptorchidism groups. Statistical significance was set at *P* < 0.05.

## 3. Results

### 3.1. The Baseline Characteristics for Groups with or without Cryptorchidism

A total of 223 CHH patients were retrospectively evaluated in this study. They were in good conditions with normal blood and urine routine test results and normal liver and renal function. Thyroid hormones, adrenal glucocorticoids, and IGF-1 levels were all in the normal range. All patients had normal pituitary MR images.

Of 40 cryptorchidism CHH patients, 25 patients (25/40, 62.5%) were diagnosed as Kallmann syndrome because they were anosmic and they had olfactory bulb aplasia in MR images. Another 15 patients were diagnosed as nCHH. The proportion of Kallmann syndrome in the noncryptorchidism group was 47.0% (86/183). The basal testicular volume in cryptorchid patients was smaller than that in noncryptorchid patients (1.6 ± 1.1 ml vs. 2.2 ± 1.6 ml, *P*=0.013). After gonadotropin treatment, the testicular size in cryptorchid and noncryptorchid patients increased to 6.2 ± 4.1 ml vs. 8.6 ± 4.6 ml, respectively, (*P*=0.003). The rate and duration of pretreatment androgen exposure were similar between cryptorchid and noncryptorchid patients. The age of initiating treatment and peak LH level after triptorelin stimulation was similar between two groups as well (see Table 1).

Several comorbidities were observed in all these CHH participants, such as obesity (*n* = 23), dwarfism (*n* = 9), mental retardation (*n* = 5), and unilateral agenesis of the kidney (*n* = 3) and cleft lip and palate (*n* = 3). The incidences of these comorbidities were similar between two groups (see Table 1).

### 3.2. Gonadotropins Boost Testosterone Level and Testicular Volume of the Patients with Cryptorchidism

For all the cryptorchid patients, the mean basal testicular volume was 1.6 ± 1.1 mL. Eighteen patients presented unilateral and 22 bilateral cryptorchidism. Twenty-three patients underwent orchidopexy at a mean age of 8.3 ± 6.0 years (ranged from 1 to 23 years). Baseline serum levels of LH, FSH, and testosterone were 0.2 ± 0.6 IU/L, 0.8 ± 0.9 IU/L, and 0.8 ± 0.5 nmol/l, respectively. Peak LH level after triptorelin stimulation was 4.5 ± 11.9 IU/L. Patients began gonadotropins treatment at the age of 21.8 ± 5.0 years. The median follow-up period of the patients was 24 (15, 33) months (totally 960 person-months). After gonadotropins treatment, their serum testosterone increased from 0.8 ± 0.5 nmol/L to 14.1 ± 8.2 nmol/L (*P* < 0.001) and the testicular volume enlarged from 1.6 ± 1.1 ml to 6.2 ± 4.1 mL (*P* < 0.001).

### 3.3. Spermatogenesis and Paternity Outcomes in CHH Patients with Cryptorchidism

**Figure 1:**
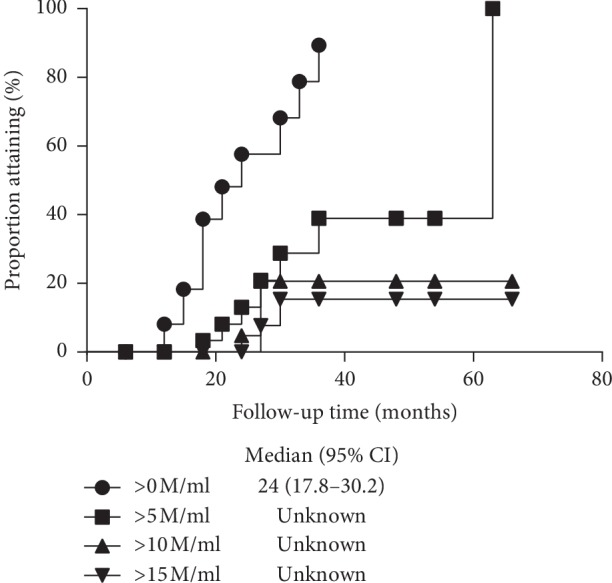
Median time for achieving sperm counts at different thresholds. Kaplan–Meier analysis of the median times for achieving sperm concentration more than 0, 5, 10, and 15 million/ml. *n* = 40.

The median time for first sperm appearance in semen of cryptorchid patients was 24 months (95% CI, 17.8–30.2, using the Kaplan–Meier analysis) after gonadotropin therapy. The estimated median time for sperm concentration >5, 10, and 15 million/ml could not be obtained due to insufficient number of patients who produced sperms above these levels (Figure 1). Orchidopexy history (*P*=0.054), unilateral or bilateral cryptorchidism (*P*=0.225), peak LH level after triptorelin stimulation (*P*=0.210), as well as basal testicular volume (*P*=0.119) were not significant predictors for spermatogenesis outcome according to Cox regression analysis (Table 2). For patients with orchidopexy (*n* = 23), age for surgery (*P*=0.448) was also not a significant contributor for successful spermatogenesis.

Of the 20 (20/40, 50%) patients who succeeded in spermatogenesis, 10 had unilateral and 10 bilateral cryptorchidism. The mean time to achieve the first sperm is 19 ± 8 months. Only 2 patients (2/20, 10%) attained lower reference limit of sperm concentrations (≥15 million/ml) according to WHO standards [16]. Five patients (5/20, 25%) had normal sperm progressive motility (*A* + *B*) (≥32%). Of the 9 patients who desired for fathering children, 5 patients (56%) (2 with unilateral and 3 bilateral cryptorchidism) impregnated their partners during the treatment. Three boys and 2 girls were born with normal external genital appearance.

### 3.4. Comparison of Spermatogenesis between CHH Patients with and without Cryptorchidism

The mean follow-up period was similar between the two groups, cryptorchidism CHH 24 (15, 33) months and noncryptorchidism CHH 23 ± 13 months (*P*=0.438). The success rate of spermatogenesis in the cryptorchidism group was lower than that of the noncryptorchidism group (50% vs. 67%, *P*=0.032) in responding to gonadotropins. Consistently, the cryptorchidism group had a longer median time for first sperm detection in semen (24 months vs. 15 months, *P* < 0.001) and a lower mean sperm concentration (1.9 (0.5, 8.6) million/ml vs. 11.1(1.0, 25.0) million/ml, *P*=0.006) during the treatment. However, sperm progressive motility (*A* + *B*) ((30.4 ± 24.0) % vs. (37.1 ± 20.6) %, *P*=0.372) and total mobility (*A* + *B* + C) ((34.8 ± 24.9) % vs. (44.6 ± 20.5) %, *P*=0.210) were similar between the two groups (Table 3).

## 4. Discussion

It has been suggested that cryptorchidism history is a detrimental risk factor for spermatogenesis, and many clinicians empirically believed that the chance of successful spermatogenesis was extremely low in cryptorchid CHH patients [17, 18]. Our present work showed that 50% (20/40) of CHH patients with cryptorchidism history succeed in spermatogenesis, and 5 patients impregnate their wives during gonadotropin therapy.

Cryptorchid CHH patients (even bilateral) possess the potentiality of spermatogenesis and impregnating their wives [19, 20]. In this study, the successful rate of producing sperms is lower in cryptorchidism patients than noncryptorchidism patients. Several factors may contribute to this phenomenon. First, for CHH patients, absence of mini puberty may block the transformation from gonocyte into adult (dark) spermatogonia in the seminiferous tubules, leading to a significant reduction in fertility potentiality [21, 22]. Second, spermatogenesis needs an optimal scrotal temperature of 33°C. Higher temperature for undescended testis directly impairs the procession of sperm maturation ^3^. Last, excessive oxidative stress and inflammatory reaction were found in undescended testis, which have negative effects on future fertility [23].

In our study, orchidopexy history, age for surgery, unilateral or bilateral cryptorchidism, as well as basal testicular volume did not correlate with spermatogenesis outcome. In order to maximally preserve the spermatogenesis capacity, it is recommended to conduct orchidopexy between 6 and 12 months of age. If the condition is diagnosed later in life, surgery should be performed at the earliest time [9, 24]. A randomized controlled study conducted by Kollin et al. [25] showed that orchidopexy performed at the age of 9 months produced a better testicular function than that performed at the age of 3 years. Earlier surgery leads to a larger number of Sertoli and germ cells and a larger testicular volume. Canavese's study [26] provided further evidence to support earlier surgery. Compared with orchidopexy performed in the second year of life, surgeries performed during the first year of life had clearly better results in total sperm counts and sperm motility. However, only 20% of cryptorchid boys in England underwent surgeries before the age of 18 months because surgeons are reluctant to conduct operations on newborns [27]. In our study, most of the participants conducted orchidopexy at the age over 2 years, possibly due to economic constraints or insufficient knowledge of the disease. It is presumed that the severely impaired testicular function could not be compensated by orchidopexy which was done too late. This may explain the finding that the age for orchiopexy had no influence on spermatogenesis in our study.

It has been reported that men undergoing bilateral orchidopexy in their childhood have poorer fertile capacity compared with those with unilateral procedure [1, 2]. The paternity rate is approximately two-thirds and less than one-third, respectively, in patients with unilateral and bilateral cryptorchidism [28]. However, our study showed no difference between these 2 groups, possibly due to small sample size and different etiology of our participants. Patients in our study are CHH with cryptorchidism, while patients in the other studies are with isolated cryptorchidism (with normal gonadotropin secretion). Furthermore, it was reported that men presenting with unilaterally undescended testis may have bilateral testicular abnormality [29].

Till now, more than two dozens of genes have been identified to cause CHH. Mutations of some genes, such as *FGFR1* and *KAL1*, may impair testicular development and spermatogenesis [30–32]. The incidence of cryptorchidism was up to 67% in patients with *KAL1* mutation [32], while 20% in patients was caused by FGFR1 mutations. One study, including 90 CHH patients treated with pulsatile GnRH infusion, showed that nearly 14% of patients had impaired testicular response to endogenous gonadotropins [33]. Therefore, the impaired testicular function in our participants may be caused by CHH-related gene mutations. As different gene mutations may markedly influence testicular function and spermatogenesis outcome, genetic screening of these patients ought to be conducted in a prospective study.

Multiple factors, such as gonadotropin deficits (i.e., absent mini puberty), temperature, oxidative stress, and age for orchidopexy, may remarkably influence the function of undescended testis. Additionally, testicular function may be impaired by surgical intervention, suggesting the surgery procedure itself is a potential confounding factor [24]. The compounding situation made it difficult to quantitatively analyze the effect of each factor on the patient's fertility outcome.

Some limitations should be addressed. First, since our conclusion is stemmed from CHH patients, it should be prudent to extrapolate the results to cryptorchidism population without CHH. Second, orchidopexy was done relatively late in our patients, which would bring potential selection bias. Third, as a retrospective study, HCG/HMG was the only regime prescribed to our participants during 2005–2014. Recent data revealed that HCG/rhFSH is superior to HCG/HMG for spermatogenesis [34, 35]. It is hopeful that HCG/rhFSH regime may further improve the outcome of spermatogenesis in CHH patients with cryptorchidism.

## 5. Conclusions

In summary, our study demonstrates that the successful rate of spermatogenesis in CHH patients with cryptorchidism is 50% and the pregnant rate is about 50% in couples desiring for children. This study provides more accurate prognostic information for CHH patients with cryptorchidism after gonadotropin treatment. Prospective studies with large cohort are needed to determine potential predictors on spermatogenesis in this population.

## Figures and Tables

**Table 1:** Comparison of baseline characteristics between the cryptorchidism and noncryptorchidism group.

	Cryptorchidism (*n* = 40)	Noncryptorchidism (*n* = 183)	*P* value
Proportion of Kallmann Syndrome	62.5% (25/40)	47.0% (86/183)	0.083
Age initiating treatment (y)	18.9 ± 4.6	20.4 ± 4.8	0.063
BMI (kg/m^2^)	21.2 ± 4.1	22.6 ± 3.7	0.027^*∗*^
Peak LH (IU/L)^#∧^	0.8 (0.4, 3.3)	1.7 (0.7, 5.5)	0.812
Rate of CHH family history	7.5% (3/40)	8.2% (15/183)	0.591
Basal testicular volume (ml)	1.6 ± 1.1	2.2 ± 1.6	0.013^*∗*^
Testicular volume after treatment (ml)	6.2 ± 4.1	8.6 ± 4.6	0.003^*∗*^
Proportion of preandrogen therapy	72.5% (29/40)	55.7% (102/183)	0.054
Duration of androgen therapy (months)^#^	17 (3, 45)	3 (3, 6)	0.061
Proportion of pre-HCG therapy	37.5% (15/40)	24.0% (44/183)	0.112
Duration of pre-HCG therapy (months)^#^	6 (0, 23)	3 (3, 5)	0.070
Comorbidities			
Obesity	10.0% (4/40)	10.4% (19/183)	0.878
Dwarfism	5.0% (2/40)	3.8% (7/183)	0.772
Mental retardation	2.5% (1/40)	2.2% (4/183)	0.583
Unilateral agenesis of the kidney	2.5% (1/40)	1.1% (2/183)	0.672
Cleft lip and palate	0.0% (0/40)	1.6% (3/183)	0.091

^*∗*^Statistical significance. *P* < 0.05 was defined as significant difference. ^#^Data were expressed as median (25%, 75%). ^∧^Peak LH: highest LH level after intramuscular injection of triptorelin 100 *μ*g.

**Table 2:** No significant predictors for successful spermatogenesis (Cox regression model).

	*β*	*P* value	95% CI	95% CI
			Lower bound	Upper bound
Orchidopexy history (0, 1)	−1.622	0.054	0.038	1.027
Unilateral or bilateral cryptorchidism (1, 2)	−0.770	0.225	0.134	1.606
Peak LH level after triptorelin stimulation	0.025	0.210	0.986	1.066
Basal testis volume	0.528	0.119	0.873	3.294

**Table 3:** Comparison of spermatogenesis between the cryptorchidism group and noncryptorchidism group.

	Cryptorchidism (*n* = 40)	Noncryptorchidism (*n* = 183)	*P* value
Spermatogenesis rate	50%	67%	0.032^*∗*^
Median time for first sperm detection in semen (months)	24	15	<0.001^*∗*^
Mean sperm concentration^#^	1.9 (0.5, 8.6)	11.1 (1.0, 25.0)	0.006^*∗*^
Sperm progressive motility (*A* + *B*) (%)	30.4 ± 24.0	37.1 ± 20.6	0.372
Sperm total mobility (*A* + *B* + *C*) (%)	34.8 ± 24.9	44.6 ± 20.5	0.210

^*∗*^Statistical significance. *P* < 0.05 was defined as significant difference. ^#^Data were expressed as median (25%, 75%).

## Data Availability

The data used to support the findings of this study are included within the article.
